# Passive surveillance of *Leptospira* infection in swine in Germany

**DOI:** 10.1186/s40813-018-0086-5

**Published:** 2018-03-27

**Authors:** Katrin Strutzberg-Minder, Astrid Tschentscher, Martin Beyerbach, Matthias Homuth, Lothar Kreienbrock

**Affiliations:** 1 IVD Innovative Veterinary Diagnostics (IVD GmbH), Albert-Einstein-Str. 5, 30926 Seelze, Germany; 2 Institute for Biometry, Epidemiology and Information Processing (IBEI), WHO Collaborating Center for Research and Training for Health at the Human-Animal-Environment Interface, University of Veterinary Medicine Hannover, Foundation, Bünteweg 2, 30559 Hannover, Germany

**Keywords:** Pig, *Leptospira*, Bratislava, Australis, Icterohaemorrhagiae, Copenhageni, Monitoring, MAT, Seropositivity, Temporal trends

## Abstract

**Background:**

As no current data are available on the prevalence of leptospiral infection in swine in Germany, we analysed laboratory data from diagnostic examinations carried out on samples from swine all over Germany from January 2011 to September 2016. A total of 29,829 swine sera were tested by microscopic agglutination test (MAT) for antibodies against strains of eleven Leptospira serovars.

**Results:**

Overall, 20.2% (6025) of the total sample collection tested positive for leptospiral infection. Seropositivity ranged between 16.3% (964) in 2011 and 30.9% (941) in 2016 (January to September only). Of all samples, 11.6% (57.3% of the positives) reacted with only one Leptospira serovar, and only 8.6% (42.7% of the positives) reacted simultaneously with two or more serovars. The most frequently detected serovar was Bratislava, which was found in 11.6% (3448) of all samples, followed by the serovars Australis in 7.3% (2185), Icterohaemorrhagiae in 4.0% (1191), Copenhageni in 4.0% (1182), Autumnalis in 3.7% (1054), Canicola in 2.0% (585), and Pomona in 1.2% (368). Modelling shows that both the year and the reason for testing at the laboratory had statistically strong effects on the test results; however, no interactions were determined between those factors. The results support the suggestion that the seropositivities found may be considered to indicate the state of leptospiral infections in the German swine population.

**Conclusion:**

Although data from passive surveillance are prone to selection bias, stratified analysis by initial reason for examination and analyses by model approaches may correct for biases. A prevalence of about 20% for a leptospiral infection is most probable for sows with reproductive problems in Germany, with an increasing trend. Swine in Germany are probably a reservoir host for serovar Bratislava, but in contrast to other studies not for Pomona and Tarassovi.

## Background

Leptospirosis is presumed to be the most widespread zoonosis worldwide [1]. It is a cause of reproductive loss in swine breeding herds and has been reported in swine from all parts of the world [2]. Endemic infections in swine herds generally remain subclinical, as do the vast majority of leptospire infections. However, when a susceptible breeding herd is infected for the first time or its immunity is compromised, considerable losses can occur due to abortion, stillbirths, weakly piglets, or infertility. Leptospires persist in the kidneys and genital tract of carrier swine and are excreted in urine and genital fluids [2].

Swine act as maintenance hosts for the serovars belonging to the Pomona and Australis serogroups [3–6], while Icterohaemorrhagiae, Grippotyphosa, and Tarassovi serogroups are among the more commonly identified incidental infections in swine [2]. Serovar Bratislava is endemic in swine in some regions [7–9]. Serological testing is the laboratory procedure most frequently used to confirm the clinical diagnosis, to determine herd prevalence, and to conduct epidemiological studies. The standard serological test is the microscopic agglutination test (MAT). The minimum antigen requirements are that the test should comprise representative strains of all the serogroups known to exist in the particular region as well as those known to be maintained elsewhere by the host species. A titre of 100 is taken as positive for the purpose of international trade [10], but given the high specificity of the MAT, lower titres can be taken as evidence of previous exposure to Leptospira. The MAT is used to test individual animals and herds. As an individual animal test, the MAT is very useful (due to its high sensitivity) for diagnosing acute infection: a four-fold rise in antibody titres in paired acute and convalescent serum samples is diagnostic. To obtain useful information from a herd of animals, at least ten animals, or 10% of the herd, whichever is greater, should be tested for a sufficient sensitivity, and vaccination history should be considered, if vaccines are available [2, 10]. In Germany no vaccine was registered as of 31 August 2016 [11] and there still is no vaccine for swine available in Germany. However, the use of imported vaccines is allowed with special permission. The MAT has limitations in the diagnosis of chronic infection in individual animals and in the diagnosis of endemic infections in the herds. Infected animals may abort or be renal/genital carriers with MAT titres below the widely accepted minimum significant titre of 100 at final dilution [2, 10]. Because of all these factors, it is not permissible to express the specificity and sensitivity of the MAT as percentages.

There are only a few recent studies on domestic swine; these report seroprevalences of 55.9% in pigs in Colombia [12], 16.1% in pigs in technified swine farms in the state of Alagoas, Brazil [13], 8.6% in pigs in Korea [14], and 2.7% in swine in Poland for selected serovars [15]. Furthermore, there are two older studies [16] that report prevalences ranging from 1.2% for pigs in Germany [17] to 73.3% in sows in Vietnam (Mekong Delta) [9].

Although leptospirosis is no longer an OIE-listed disease, leptospirosis in swine and sheep is still classified as a notifiable disease and a zoonosis in Germany, but there are no current German data available on this infection in swine. The latest current data in Germany were collected in 1984 and reported in 1987 [17]. Data from a passive surveillance of swine in Germany for infection with leptospires (2003 to 2010) were presented at the EuroLepto 2012 [18].

For this paper we have analysed routine laboratory data from January 2011 to September 2016 for the seropositivity of a total sample collection and subcollections based on the reason for the examination with the aim to estimate the extent of infection of swine in Germany with leptospires and to identify the occurring serovars or serogroups.

## Methods

### Sample collection

Diagnostic examinations were carried out at the diagnostic laboratory of IVD GmbH, Seelze, Germany, on 29,829 serum samples collected from swine from all over Germany between January 2011 and September 2016. All available information about the serum samples, e.g. farm of origin, age/gender of the animal source, was collected with a lab information system (Ticono-LC, Ticono GmbH, Hannover, Germany) and taken into consideration if sufficient information was available. The frequency of samples sent for examination according the geographic origin in Germany was parallel to the density of swine husbandry in Germany (data from further analyses, not published). Samples came from 2571 animal owners for the total study period. We furthermore analysed the samples per herd and per year. Since some farms sent samples for examination in more than one year, the sum of farms sending samples per year is 3953. It is very likely that most of the samples were from animals kept indoors in stables, because less than 1% of swine in Germany are housed outdoors, but this was not explicitly reported. And since more than 99% of swine were housed indoors and no climatic data within the stables were available, seasonal aspects could not be analysed here seriously.

As there was very little or no further information about the sows (such as parity), this was therefore not taken into account for the analyses.

Preliminary information about the samples, such as clinical symptoms or reason for examination, had been systematically requested with the submission form of the laboratory (IVD GmbH, Seelze, Germany) and collected with the Ticono-LC lab information system, but not all senders filled the form out completely. Available information about the samples about the reason for examination was used to identify two subcollections which were analysed in comparison with the total sample collection.

The subcollection “reproductive problems” comprised all samples (n = 12,017) of the total population for which any reproductive problem had been reported (checkbox “Reproductive symptoms and/or any comment about reproductive problems” in the form). The subcollection “Monitoring” comprised all samples (n = 1813) of the total sample collection for which the checkbox “Examination for monitoring reasons (no clinical symptoms, health check)” was marked and no reproductive problem had been reported.

### Laboratory methods

All samples were tested for leptospiral antibodies by the microscopic agglutination test (MAT) according to the OIE Manual of Diagnostic Tests and Vaccines for Terrestrial Animals 2008 [19] to current editions 2014 [10] using live antigens of Leptospira serovars Australis (strain Ballico), Bratislava (strain Jez Bratislava), Canicola (strain Hond Utrecht IV), Grippotyphosa (strain Moskva V), Copenhageni (strain M20), Icterohaemorrhagiae (strain RGA), Pomona (strain Pomona), Hardjo (strain Hardjoprajitno), Saxkoebing (strain Mus 24), and Tarassovi (strain Perepelitsin). In response to an analysis of the frequencies of seropositivity of Leptospira serovars worldwide [16], the serovar Saxkoebing was replaced with Sejroe (strain M 84) in February 2011, and the serovar Autumnalis (strain Akiyami A) was added in April 2011. All strains were supplied by the Leptospirosis Reference Laboratory (at KIT Biomedical Research, The Netherlands). Sera were pretested at the final dilution of 1/100. Sera with 50% agglutination were retested to determine an endpoint using dilutions of sera beginning at 1/25 through 1/3200. Serum samples with the widely accepted minimum significant titre of 100 (reciprocal of the final dilution of serum with 50% agglutination) were assessed positive. A farm was considered positive for leptospiral infection if at least one sample per year tested positive by MAT.

### Statistical methods

Data were analysed in two steps. For a general overview, all data were first analysed independently; the positive findings then were analysed both generally (Table 2) and by serovar (Tables 3 and 4). The results are reported as usual frequency statistics.

Next, in order to take into account the hierarchical data structure (repeated samples per farm), all data were analysed in a hierarchical, logistic regression model with two fixed regressors (“year” and “reason for sampling and testing”) and a random factor (“farm”) (Table 5). From this the strength of association between seropositivity and these factors was estimated via odds ratios (OR) and the asymptotic 95% confidence intervals (CI) of Woolf and the associated likelihood ratio test (Table 6). All analyses were performed using SAS, version 9.3 TS level 1 M2 (SAS Institute Inc., Cary, NC, United States).

## Results

### General description of the sample population

In general, 29,829 samples from routine laboratory diagnostics were examined from January 2011 to September 2016. Data are from 2571 different farms with some repeated analyses from year to year. These multiple submissions yielded a sum of 3953 farms. Most of the farms (53.8%) sent four to nine samples per year, followed by 19.0% of farms with only one to three samples per year, and 18.8% of farms sending ten to 14 samples per year. The reason for laboratory analysis for each sample is indicated in Table 1. Of the samples for which a reason was given for the examination (*n* = 13,830), 86.9% were sent with the information “reproductive problems”; only 13.1% of the samples were designated as having been taken for “Examination for monitoring reasons (no clinical symptoms, health check)” without any reported reproductive problem.

**Table 1:** Collection of swine serum samples tested for leptospires by MAT

	Reasons for testing of individual samples			Total	Number of farms
	Monitoring	Reproductive problems	Unknown reasons		
2011	392	2514	3002	5908	856
2012	400	2422	2650	5472	797
2013	338	2249	2981	5568	718
2014	298	2087	2721	5106	659
2015	300	1582	2851	4733	529
2016 / 01 to 09	85	1163	1794	3042	394
Total	1813	12,017	15,999	29,829	3953^1^

^1^Because some farms sent samples in more than one year, this number of farms is higher than that of the different farms tested in the total study period

Overall, there was no information at all about the animal source for 42.2% of all samples (*n* = 12,600), but there was information for 57.8% (*n* = 17,229) of the total sample collection. Of the samples with information about the animal source, 95.9% (*n* = 16,529) were from sows (sows and gilts). (Data not shown).

Only 1813 samples from the entire sample population, i.e. 6.1%, were identified as having been taken due to monitoring. Overall, the sample population is therefore dominated by samples from sows with reproductive problems. Most of the samples are from farms in Northwest Germany, which is the center of German swine production. All in all, the serological findings do not correspond to a regular prevalence, because the sample collection was not a cross-sectional study from the entire German swine production. Nevertheless, the data do give insight into the *Leptospira* occurrence in German pig farms, because the study included a substantial collection of German pig breeding farms. According to figures from the Federal Statistical Office [20], this represents a mean of 5.9% of the officially registered German breeding farms per year.

### General seropositivity

A general overview of the results of the serological testing is reported in Table 2. Overall, 20.2% (n = 6025) of all 29,829 swine serum samples tested positive by MAT. The seropositivity ranged between 16.3% (n = 964) in 2011 and 30.9% (n = 941) in 2016 (January to September). A total of 64.5% farms tested positive for leptospires by at least one sample per year. The percentage of farms with positive test results ranged between 59.3% (n = 508) in 2011 and 78.2% (n = 308) in 2016. Forecasting 4056 samples for all of 2016, the mean number of samples was 5141 per year, with moderate variation in seropositivity from year to year (Table 2).

**Table 2:** Results of swine serum samples tested for leptospires by MAT. Number (n) of samples examined, numbers (n) and percentage (%) of positive samples and farms with at least one positive sample per farm and year from January 2011 to September 2016, and totals

Year	Number of serum samples	Positives^a^		Number of farms	Positives^b^	
		n	%		n	%
2011	5908	964	16.3	856	508	59.3
2012	5472	1042	19.0	797	509	63.9
2013	5568	1076	19.3	718	434	60.4
2014	5106	993	19.5	659	416	63.1
2015	4733	1009	21.3	529	375	70.9
2016 / 01 to 09	3042	941	30.9	394	308	78.2
Total	29,829	6025	20.2	3953	2550	64.5

^a^Samples with a titre ≥100

^b^farms with at least one positive sample per year

Analysis of the reactivity of the serum samples with different serovars (Table 3) showed that 11.6% of all samples examined, comprising 57.3% of the positives, reacted with only one serovar, whereas 42.7% reacted simultaneously with two or more serovars.

**Table 3:** Simultaneous reactivity of swine serum samples by MAT with various *Leptospira* serovars. (Data from January 2011 to September 2016)

Reactivity with different serovars (number of serovars)	Number of serum samples	Percentage of all samples
0	23,804	79.8
1	3454	11.6
2	1617	5.4
3	524	1.8
4	201	0.7
5	95	0.3
6	69	0.2
7	41	0.1
8	16	0.1
9	7	0.0
10	1	0.0
Total	29,829	100.0

### General occurrence of serovars and variations from year to year

The most frequently detected serovar was Bratislava (Table 4), which was found in 11.6% (*n* = 3448) of all samples, followed by the serovars Australis in 7.3% (*n* = 2185), Icterohaemorrhagiae in 4.0% (*n* = 1191), Copenhageni in 4.0% (*n* = 1182), Autumnalis in 3.7% (*n* = 1054), Canicola in 2.0% (*n* = 585), and Pomona in 1.2% (*n* = 368). All other serovars were detected less often (in < 1.0% of all samples). Total reactivity is more than 100.0% because of the possibility of multiple positive reactions with different serovars.

**Table 4:** Number and percentage of swine serum samples that tested positive by MAT for *Leptospira* serovar. (Data from January 2011 to September 2016)

Serogroup	Serovar	Number of positive serum samples	Percentage of the positives (samples tested positive in total: 6025)	Percentage of all tested (samples tested in total: 29,829)
Australis	Australis	2185	36.3	7.3
	Bratislava	3448	57.2	11.6
Autumnalis	Autumnalis^a^ (tested: 28,189, positive: 5735)	1054	18.4	3.7
Canicola	Canicola	585	9.7	2.0
Grippotyphosa	Grippotyphosa	230	3.8	0.8
Icterohaemorrhagiae	Copenhageni	1182	19.6	4.0
	Icterohaemorrhagiae	1191	19.8	4.0
Pomona	Pomona	368	6.1	1.2
Sejroe	Hardjo	35	0.6	0.1
	Sejroe^b^ (tested: 29,247, positive: 5902)	9	0.2	0.0
Tarassovi	Tarassovi	151	2.5	0.5

^a^Tested since April 2011

^b^Tested since February 2011

### Trends in seropositivity in time and subcollections

This model (Table 5) shows a strong, statistically significant effect of the year of analysis (general p = < .0001). Starting in 2011 as a reference, the seropositivity was statistically significantly increased in 2012, 2015, and 2016. In addition, the reason for laboratory analyses influenced the results (general p = 0.0005): in contrast to samples from monitoring, those from animals with reproductive problems were 1.5 times more likely to be positive. Due to the same order of effect for samples of unknown reason, it may be inferred that those were from sows with reproductive problems, as well. However, no interaction was found between year and reason for sampling (p = 0.1049), which supports the suggestion that there was a real expansion of Leptospira in the German swine population. These models support the evidence that Leptospira infections increased over time.

**Table 5:** Two-factor logistic regression analysis on MAT outcome

General model type III tests of fixed effects						
	DF^a^		F^b^		*p* ^c^	
Year	5		10.79		< .0001	
Reason for sampling	2		7.66		0.0005	
Interaction	10		1.58		0.1049	
Effect	n	Positives %	OR^d^	CI^e^		*p* ^f^
				lower	upper	
Year						
2011 (ref)	5908	16.3	1	–	–	–
2012	5472	19.0	1.490	1.204	1.843	0.0023
2013	5568	19.3	1.251	0.996	1.572	0.1292
2014	5106	19.5	1.296	1.026	1.638	0.1935
2015	4733	21.3	1.951	1.549	2.457	0.0014
2016 / 01 to 09	3042	30.9	2.565	1.862	3.533	0.0240
Reason for sampling						
Monitoring (ref)	1813	16.9	1	–	–	–
Reproductive problems	12,017	22.1	1.478	1.189	1.838	0.0017
Unknown reasons	15,999	19.1	1.326	1.068	1.646	0.0093

^a^Degrees of freedom

^b^F-test statistics for model parameter

^c^*p*-value for F-Test

^d^odds ratios adjusted for year, reason for sampling, and interaction

^e^lower/upper bound 95% confidence interval for the odds ratio

^f^*p*-value for Wald test

To compensate for a selection bias in these analyses, trends in time were analysed separately by means of the logistic regression in the stratum of reason for sampling (see Table 6).

**Table 6:** One-factor logistic regression analyses on MAT outcome. Stratified by reason for sampling

Effect	number	Positives %	OR^a^	CI ^b^		p^c^
				lower	upper	
Reason: monitoring						
2011 (ref)	392	9.2	1	–	–	–
2012	400	20.5	2.450	1.386	4.331	0.0022
2013	338	15.1	1.623	0.876	3.007	0.1231
2014	298	15.8	1.538	0.818	2.895	0.1806
2015	300	23.7	2.768	1.496	5.123	0.0013
2016 / 01 to 09	85	23.5	2.840	1.161	6.948	0.0224
Reason: reproductive problems						
2011 (ref)	2514	18.0	1	–	–	–
2012	2422	18.3	1.051	0.863	1.280	0.6223
2013	2249	20.5	1.071	0.874	1.312	0.5075
2014	2087	23.8	1.326	1.081	1.627	0.0068
2015	1582	27.6	1.679	1.355	2.080	<.0001
2016 / 01 to 09	1163	32.0	2.301	1.822	2.906	<.0001
Reason: unknown						
2011 (ref)	3002	15.9	1	–	–	–
2012	2650	19.6	1.284	1.055	1.562	0.0127
2013	2981	18.9	1.131	0.928	1.377	0.2233
2014	2721	16.5	1.076	0.878	1.319	0.4813
2015	2851	17.6	1.603	1.296	1.983	<.0001
2016 / 01 to 09	1794	30.6	2.585	2.078	3.215	<.0001

^a^Odds ratios adjusted for year, reason, and interaction

^b^lower/upper bound 95% confidence interval for the odds ratio

^c^*p*-value for Wald test

The results (Table 6) show time effects very similar to those in the general model from Table 5, indicating the presence of a general trend over time. This effect is different in the monitoring group in contrast to both the stratum with reproduction problems as well as in the subgroup with unknown reason for sampling. Nevertheless, both trends are very similar, indicating that it is likely that most of the unknowns are due to reproductive problems, as well.

## Discussion

Because research results on the incidence of leptospire infections in domestic pigs are either old, scarce, or both, this study used existing diagnostic data in order to obtain current evidence about the infection of swine with leptospires in Germany. The advantage of the present approach is that it yielded a large number of results. Of course, the disadvantage of this approach is that the sample collection is not representative. In light of biases due to the structure of data from routine diagnostic examinations, logistic regression analyses were undertaken including additional information about the farm and stratification by the reason for testing; in this way the biases could be compensated for, and it was shown that the seropositivities of the present study are plausible estimations for the occurrence of *Leptospira* in the German swine population.

Available epidemiological studies about leptospirosis in swine are very heterogeneous, due to regional differences and to differences in the evaluation of diagnostic (e.g. different serovars used for testing) and population studies. In the collection of our investigation we found an overall seropositivity of 20.2%, with an increasing trend over time. A similar seroprevalence of 16.1% [13] has been found in 342 pigs in five districts in the state of Alagoas, Brazil, but in contrast to our results the most frequent serovar there was Icterohaemorrhagiae (41.8% of the 55 positives), followed by Autumnalis (29.1%) and Bratislava (9.1%), which was the most frequent serovar in our present study (57.2% of positives).

A recent study about the prevalence of antibodies to selected *Leptospira* serovars in swine from Poland (*n* = 22,883) showed prevalences of only 1.32% to 2.68% within the very similar time period of 2011 to 2015; there, the most frequent serovars were Pomona (varying between 0.39% to 1.13%) and Sejroe (decreasing from 1.12% to 0.18%), but it was not tested for antibodies against the serovar Bratislava in that study [21]. In our study, seroprevalences for Pomona were slightly higher, while those for Sejroe were much lower. Results show that, even in geographically similar or close regions, overall prevalences of *Leptospira* infections may be similar, while the frequency of serovars may vary substantially. Differences in the frequency of serovars may be additionally caused by infection dynamics over periods of time due to the population density of the wildlife reservoir hosts and climatic conditions (mean air temperature ≥ 18 °C and periods of heavy rain).

More than half (57.3%) of the positive porcine sera reacted by MAT with a single serovar, presumably indicating the infection causing serovar and the chronic stage of infection, so that ultimately it is to be assumed that 11.6% of the examined pigs were chronically infected by *Leptospira* [22]. On the other hand, 42.7% of the positive porcine sera reacted simultaneously with two or more serovars, indicating both cross-reactions of serovars of the same serogroup and the acute phase of infection, because of the induction of antibodies against common antigens of *Leptospira* in the first phase of infection. Moreover, paradoxical immune response occurs in the state of acute infection, meaning that 3.2% of all pigs examined in this investigation were very likely in an acute stage of infection (reactions with three or more serovars simultaneously, as two serovars at most belong to one serogroup) [22]. Considering that the MAT is a not a perfect test, in that it is highly specific, but of low sensitivity in case of chronic and endemic infections [10], the seropositivities of this study are very likely underestimated.

Because of the different epidemiology of the serovars or serogroups, each serovar tested in this study will be discussed separately:

On the basis of these results and those of earlier analyses [18], **Bratislava** is apparently still endemic in pigs in Germany, where pigs are a reservoir host for this serovar, as is the case in many countries and regions worldwide [16]. As venereal transmission is thought to play an important role in the spread of Bratislava infection, this is the most critical factor in control of the infection. Nevertheless, because of the difficulties in culturing these strains [17] and the inability thus far to identify the serovar by detection of leptospires via PCR techniques, Bratislava infections of swine still remain poorly understood [2].

Although the reported percentages of the positives for serovar **Pomona** are in general not higher than 6.5% (6.1% in this study), with a few exceptions [12, 23] Pomona in particular is nevertheless still found in cases with documented abortions in Germany [24]; unfortunately no newer data are available. The diagnostic services of IVD GmbH, Seelze, Germany, reported a few cases of abortions per year with strong indication that these were caused by Pomona (personal communication). Because of these occasional outbreaks of clinical disease in contrast to widespread clinical disease of swine-adapted strains it is assumed that these strains are rodent maintained [2, 25, 26].

The pig was previously thought to act as a maintenance host for some strains of the serovar **Tarassovi** found in eastern Europe and Australia [2], but declining seroprevalences in most of the studies [16, 21] as well as values of 0.5% observed overall in this study support the view that Tarassovi infections are incidental infections of pigs resulting from wildlife contact and that the swine is no longer a reservoir for this serovar.

Although leptospires belonging to the serogroup **Canicola** have been recovered from swine in a number of countries[2, 27], little is known about the epidemiology of serovar Canicola infection in pigs. Dogs are recognized as the maintenance host for this serovar but wildlife may also be a source of infection. Long periods of urine shedding observed in infected pigs and the ability of Canicola to survive in undiluted pig urine suggest that intraspecies transmission occurs [2]. Although seroprevalences for serovar Canicola worldwide in general are not higher than 1.5% [9, 16], it was nevertheless the most frequent serovar in a study on an area of the Colombian tropics in pigs (62.4%) and in humans (64.5%), and less frequent in dogs (14.1%) [12]. Furthermore, an overall seroprevalence of 2.0% with a continuous, statistically significant increase over time was observed in the present study. Because of generally high biosecurity levels in German pig herds, it is unlikely that pigs could be infected with serovar Canicola via dogs or wildlife. Further studies should therefore be performed to investigate the entry of serovar Canicola into pig herds in Germany.

Serological evidence of **Icterohaemorrhagiae** serogroup infection has been reported in many countries with different frequencies [16], but few isolations have been made from pigs [2]. Both serovars Copenhageni and Icterohaemorrhagiae may be involved, as is supported by our data with overall seropositivities of 4.0% and 4.0%, corresponding to 19.6% and 19.8% of the positives, respectively. It is probable that both serovars were introduced to susceptible herds via an environment contaminated with urine from the infected brown rat *(Rattus norvegicus),* which is the maintenance host for these serovars. Rodents have been suspected in infections with Icterohaemorrhagiae in swine farms in Brazil, as well [13].

Serovar **Grippotyphosa** infection is maintained by wildlife hosts, and incidental infection of pigs gives rise to low prevalences in some regions, particularly in eastern and central Europe and the United States [2], as also observed in the present study (0.8% overall). A human *Leptospira* outbreak with serovar Grippotyphosa among strawberry pickers in Germany in the summer of 2007 was in all likelihood due to transmission via field mouse *(Microtus arvalis)* [28]. Depending on appropriate climatic conditions for leptospires and the population density of these mice, there is a risk of infection with leptospires via field mouse on any farm.

The seropositivity of serovar **Hardjo** in pigs of this study was very low (0.1%; 35/29829), whereas it was the most frequent serovar (3.1%) in wild boars in Poland [29]. Furthermore, serovar Sejroe, which like serovar Hardjo belongs to the serogroup **Sejroe**, was very rarely detected serologically (0.0%; 9/29247) in this study, whereas it was the most common serovar in swine in Poland, although at a low level of prevalence (1.12% for 2011 to 0.18% for 2015) [15]. Serovar Hardjo is maintained worldwide by cattle, and infection of pigs with Hardjo occurs where cattle and pigs come in close contact. Simultaneous husbandry of cattle and swine has become rare in Germany, so the risk of an infection with serovar Hardjo for pigs is low in that country, as is supported by the data of this study (seropositivity of 0.1%).

Analysis of the literature showed serious seroprevalences of serovar **Autumnalis** in pigs worldwide [16]. Results of this study, showing overall seropositivities of 3.7% with an increasing trend, confirm the need for testing of swine for antibodies against serovar Autumnalis, for which rodents or other wildlife are presumed to be the main reservoir.

Overall, interruption of transmission from infected pig or other host to the pig remains the critical factor in the control of leptospires.

## Conclusion

Infection of pigs with leptospires is obviously a dynamic process. Analyses of data from passive surveillance within routine diagnostic examinations are useful for obtaining an indication of the distribution of *Leptospira* and their serovars in domestic swine in a region in general and over time. Information about the reason for examination and the farms or herds (size and type) and animal (age and gender, clinical status) at diagnostic testing would enable an improvement in epidemiological analysis which could also be helpful for the swine veterinary practitioner. Logistic regression model approaches may compensate for the biases arising from passive surveillance data (hierarchical structures of farms, sampling strategy). Finally, this is an appeal to every swine veterinary practitioner to provide all available information about the animals and the herd in order to enable a good diagnosis and improve epidemiological analyses.

Based on these results, further active surveillance of swine in Germany for infections with leptospires should be advised. Attention should furthermore also continue to be paid to subclinical leptospiral infections as well as leptospirosis in breeding pigs and in proliferation of pigs together with their hosted infectious agents. It is strongly recommended to maintain awareness about subclinical leptospiral infections and leptospirosis in conjunction with the handling of pigs by animal owner, stockman, veterinarian, etc. However, any human, from dog owner to aquatic sportsman, can also be exposed to many other infectious sources, including other reservoir hosts (e.g. field mouse, dog) and water and soil contaminated with leptospires under optimal climatic conditions.

For better comparison of epidemiological studies about seroprevalences of leptospiral infection in swine, the serovars Bratislava, which has emerged as the major swine-maintained leptospiral infection, and Pomona, which causes clinical disease, should always be included in the examinations by MAT. It is also recommended that further serovars, like Icterohaemorrhagiae, Copenhageni, Autumnalis, Grippotyphosa, and Canicola, which are involved in incidental infections in swine by maintenance in other animal species, be included in the serovar collection of MAT for pigs in Germany and perhaps even in bordering countries or regions with similar pig management structures, because of the easy transmission by rodents and other wildlife close to swine husbandry facilities.
